# Quantitative evaluation of the progressive wear of powered interproximal reduction systems after repeated use: An in vitro study

**DOI:** 10.1007/s00056-019-00200-x

**Published:** 2019-11-13

**Authors:** C. Livas, T. Baumann, S. Flury, N. Pandis

**Affiliations:** 1 Department of Orthodontics, Academic Centre for Dentistry Amsterdam (ACTA), University of Amsterdam and VU University Amsterdam, Gustav Mahlerlaan 3004, 1081 LA Amsterdam, The Netherlands; 2 Department of Orthodontics and Dentofacial Orthopedics, Dental School/Medical Faculty, University of Bern, Freiburgstraße 7, 3010 Bern, Switzerland; 3 Department of Restorative, Preventive and Pediatric Dentistry, Dental School/Medical Faculty, University of Bern, Freiburgstraße 7, 3010 Bern, Switzerland

**Keywords:** Dental high-speed technique, Surface roughness, Optical profilometer, Dental enamel, Enamel stripping methods, Zahnmedizinische Hochgeschwindigkeitstechnik, Oberflächenrauigkeit, Optische Profilometrie, Zahnschmelz, Methoden zur Schmelzreduktion

## Abstract

**Purpose:**

To evaluate the residual surface roughness of 5 common diamond-coated interproximal reduction (IPR) systems after consecutive in vitro applications in relation to system, diamond grain size, and instrument thickness.

**Methods:**

IPR was performed on 80 extracted human incisors using motor-driven strips and discs under predefined conditions. The IPR auxiliaries were applied at 5 consecutive sessions of 20 s on intact interproximal surfaces, and the surface profile (R_a_, R_z_, R_max_) was analyzed at baseline and after each session with an optical profilometer.

**Results:**

No overall significant difference in the roughness values was found between systems (*P* = 0.07 for R_a_, *P* = 0.33 for R_z_, and *P* = 0.48 for R_max_). There was a significant average decrease of R_a_, R_z_, and R_max_ for all systems for every unit increase in time by −0.171 μm (*P* < 0.001), −3.297 (*P* ≤ 0.001), and −2.788 μm (*P* = 0.001), respectively. R_a_, R_z_, and R_max_ values increased significantly, i.e., by 0.194 μm (*P* = 0.003), 5.890 μm (*P* = 0.001), and 5.319 μm (*P* = 0.010) as instrument thickness increased by one unit. No significant reductions in R_a_, R_z_, and R_max_ were observed across grain sizes (−0.008 μm [*P* > 0.05], −0.244 μm [*P* > 0.05], and −0.179 μm [*P* > 0.05], respectively). There was no evidence of interaction between system and time as the *P* values for R_a_, R_z_, and R_max_ were 0.88, 0.51, and 0.70, respectively.

**Conclusions:**

All IPR materials presented significant gradual decrease of surface roughness after repeated applications. There were no significant roughness changes among auxiliaries of different grain sizes. Thinner auxiliaries showed significantly more roughness reduction, possibly requiring more frequent replacement than thick auxiliaries in clinical practice.

## Zusammenfassung

**Ziel:**

Beurteilt werden sollte die verbleibende Oberflächenrauigkeit von 5 handelsüblichen diamantbeschichteten Interproximal-Reduktionssystemen nach wiederholter In-vitro-Anwendung, differenziert wurde dabei bezüglich System, Diamantkorngröße und Instrumentendicke.

**Methoden:**

Interproximal-Reduktion (IPR) wurde mit motorgetriebenen Streifen oder Scheiben an 80 extrahierten menschlichen Inzisiven unter standardisierten Bedingungen durchgeführt. Die IPR-Systeme wurden für 5 aufeinander folgende Anwendungen von 20 s an intakten Interproximalflächen eingesetzt, die Oberflächenrauigkeit (R_a_, R_z_, R_max_) wurde sowohl initial als auch nach jeder Anwendung mit einem optischen Profilometer analysiert.

**Resultate:**

Es wurden keine signifikanten Unterschiede der Oberflächenrauigkeit zwischen den unterschiedlichen Systemen festgestellt (*p* = 0,07 für R_a_, *p* = 0,33 für R_z_, *p* = 0,48 für R_max_). Eine signifikante durchschnittliche Abnahme von R_a_, R_z_ und R_max_ von −0,171 μm (*p* < 0,001), −3,297 μm (*p* ≤ 0,001) bzw. −2,788 μm (*p* = 0,001) nach jeder Anwendung wurde gefunden. R_a_, R_z_ und R_max_ Werte nahmen signifikant zu um 0,194 μm (*p* = 0,003), 5,890 μm (*p* = 0,001) bzw. 5,319 μm (*p* = 0,010) bei zunehmender Instrumentendicke pro Einheit. Bezüglich der Diamantkorngrößen wurden keine signifikanten Unterschiede von R_a_, R_z_ und R_max_ (−0,008 μm [*p* > 0,05], −0,244 μm [*p* > 0,05] bzw. −0,179 μm [*p* > 0,05]) gefunden. Es gab keine Anzeichen für eine Interaktion zwischen Systemtyp und Anwendungsanzahl für R_a_, R_z_ und R_max_, mit *p*-Werten von 0,88, 0,51 bzw. 0,70.

**Schlussfolgerungen:**

Alle IPR-Systeme zeigten eine signifikante graduelle Abnahme der Oberflächenrauigkeit nach wiederholten Anwendungen. Es gab keine signifikanten Rauigkeitsdifferenzen zwischen Instrumenten mit unterschiedlichen Diamantkorngrößen. Bei dünneren Instrumenten gab es signifikant höhere Rauigkeitsverluste, was möglicherweise in der Praxis einen häufigeren Wechsel als bei dickeren Instrumenten nötig macht.

## Introduction

Space gaining procedures, e.g., tooth extractions, arch expansion, and reshaping of interproximal enamel surfaces (i.e., interproximal reduction [IPR]) are commonly applied in clinical orthodontics. Since the original introduction of IPR [1], several authors [2–6] have described in detail IPR indications and protocols for handheld or handpiece-mounted enamel cutting instruments. Overall, IPR has been used to address arch length discrepancies, to enhance anterior esthetics and interocclusal relationships, and to improve long-term stability of the treatment outcome [7].

The residual enamel roughness [8–10], and especially, the increased susceptibility to caries in vitro [11–13] initially discouraged clinicians from performing IPR in everyday practice. This perception has been drastically changed in recent years with the best available evidence indicating that IPR does not increase the incidence of caries on treated teeth [14]. Moreover, regardless of the stripping method used (i.e., abrasive strips, tungsten carbide burs or oscillating perforated diamond discs), finishing with Sof-Lex polishing discs can yield smoother surfaces than intact enamel [15].

While most of the research focused on post-IPR enamel effects, very little has been published so far on the wear of IPR materials after multiple uses [16]. Such information may have direct clinical implications since the particle size of the abrasive determines the amount of enamel reduction as well as the necessary time for polishing [17]. Lione et al. [16] demonstrated by means of tribological testing a 60% decrease in the abrasive capacity of motor-driven strips after 5 min of in vitro use, whereas at the same time almost complete detachment of diamond abrasive grains was observed by scanning electron microscope in three patients receiving IPR on mandibular incisors.

Given the growing acceptance of IPR as a minimally invasive procedure by dentists and orthodontists [18], and the widespread use of aligner treatment in combination with IPR [19], it would be interesting from a clinical point of view to investigate the surface changes on contemporary IPR materials over time. Thus, the aims of this study are to assess the roughness changes of 5 popular diamond-coated IPR systems after consecutive in vitro applications in relation to system, diamond grain size, and instrument thickness. The null hypothesis is that there is no difference in the outcome between any of the parameters.

## Materials and methods

Eighty extracted human permanent incisors with macroscopically intact interproximal surfaces, free of caries and restorations were collected from the undergraduate clinic of the Department of Preventive, Restorative, and Pediatric Dentistry, Dental School/Medical Faculty, University of Bern, Bern, Switzerland. Before extraction, patients had been informed about the use of the teeth for research purposes and verbal consent had been obtained. After extraction, the teeth were pooled. The local ethics committee categorizes pooled teeth as an “irreversibly anonymized biobank” and thus, no previous ethical approval was needed. The incisors to be used were cleaned under tap water with a scaler to remove debris and then stored in 2% chloramine solution in a refrigerator (4 °C) until needed. The incisors were then mounted in cylindrical stainless steel molds with self-curing acrylic resin (Paladur, Heraeus Kulzer, Hanau, Germany). After curing of the acrylic resin, the stainless steel molds were removed and the embedded incisors were stored in the refrigerator at 100% humidity. For the IPR procedures, the embedded incisors were then randomly allocated to 16 groups of 5 teeth each.

### IPR procedures

Five IPR systems, namely 16 instruments, were considered for the purposes of the study: DiaStrip (DentaSonic, Cham, Switzerland), Intensiv Ortho-Strips System (Intensiv SA, Montagnola, Switzerland), G5-ProLign (SDC Switzerland SA, Bioggio, Switzerland), Galaxy IPR Diamond Discs (Ortho Technology®, Lutz, FL, USA), and OS Discs (Komet USA, Rock Hill, SC, USA). The technical characteristics of IPR auxiliaries are summarized in Table 1.

**Table 1:** Technical details of the interproximal reduction (IPR) instruments tested in the study

System	Manufacturer	Instrument coding	Thickness (mm)	Particle size (μm)	Handpiece	Manufacturer
DentaSonic Diastrip	Alpin Orthodontics, Lucerne, Switzerland	DS-25	0.15	25	DentaSonic water cooling HP	Alpin Orthodontics, Lucerne, Switzerland
		DS-40	0.20	40		
		DS-60	0.30	60		
Ortho-Strips System	Intensiv SA, Montagnola, Switzerland	OS-25	0.15	25	Intensiv Swingle Reciprocating Contra Angle (WG-69 A)	W&H, Bűrnmoos, Austria
		OS-40	0.20	40		
		OS-60	0.25	60		
SDC-G5-Prostrip	SDC Switzerland SA, Bioggio, Switzerland	SDC-15	0.15	15	Ti-Max X55	NSK-Nakanishi Inc., Kanuma, Japan
		SDC-20	0.20	30		
		SDC-30	0.30	40		
Galaxy IPR Diamond Discs	Ortho Technology®, Lutz, FL, USA	OT-11	0.19	64	KaVo GENTLE-power LUX 10LP Straight 1:2	KaVo Dental, Charlotte, NC, USA
		OT-13	0.19	64		
		OT-55	0.20	46		
		OT-56	0.20	46		
OS Segment Discs	Komet USA, Rock Hill, SC, USA	OS-10	0.20	57	Komet OS 31	W&H, Bürnmoos, Austria
		OS-20	0.20	25		
		OS-18	0.18	49		

IPR was carried out by the same operator (first author) according to the manufacturers’ recommendations. For Galaxy IPR Diamond Discs, the straight handpiece was operated at 5000 rpm; for the rest of the IPR systems, the contra-angle handpieces were operated at 40,000 rpm with water cooling. The tested auxiliaries underwent five consecutive IPR sessions on intact proximal surfaces. To reproduce the average clinical treatment time, IPR sessions were set at 20 s [20, 21], reaching 100 s in total use for each auxiliary. After completion of IPR procedures, all systems were cleaned thoroughly with distilled water.

### Surface roughness evaluation

The surfaces of IPR auxiliaries were analyzed with an optical profilometer (FRT MicroProf® 100, equipped with a H0 sensor, Fries Research & Technology, Bergisch Gladbach, Germany). Linear traces were recorded at a pixel density of 1000/mm. Due to the different forms of the instruments, different total lengths of the traces were obtained. For the DiaStrip system, the Intensiv Ortho-Strips system, and the G5-ProLign system, the whole abrasive part could be measured. The resulting trace lengths were 13 mm (DiaStrip and Intensiv Ortho-Strips) and 17 mm, respectively (G5-ProLign). The sector-shaped OS Discs were measured at the outer edges of the discs, where trace lengths of 5 mm could be obtained. For the disc shaped Galaxy IPR Diamond Discs, traces were measured radially from the outer edges toward the center. For three of the discs, namely OT–11, OT–13, and OT–55, radial traces through the whole abrasive part could be obtained in that way. The trace lengths were 2.7 mm (OT–11), 2.5 mm (OT–13), and 5.2 mm (OT–55). For the fourth disc, OT–56 containing a perforated surface, not the whole abrasive surface could be measured, as there was no radial linear path through it. We nevertheless obtained 3.7 mm long traces for this disc type. The average surface roughness (R_a_; in μm), the maximum roughness depth (R_max_; in μm), and the arithmetic mean height of the surface profile (R_z_; in μm) where then determined for all the traces measured with a special software (Mark III, Fries Research & Technology GmbH, Bergisch-Gladbach, Germany). Profilometric measurements were performed at baseline, i.e., before initiating IPR (T0), and after each session, i.e., at 20, 40, 60, 80, and 100 s (T1–T5) by a second examiner (third author), blinded to the experimental groups.

### Statistical analysis

Random effects linear regression models were fitted using R_a_, R_z_, and R_max_ as the dependent variables respectively and system, grain size, thickness, and time. Interactions between system and time were also assessed. The level of statistical significance was set at 5%. Statistical analysis was conducted with the Stata Statistical Software (Release 15, StataCorp LLC, College Station, TX, USA).

## Results

The surface roughness values (R_a_, R_z_, R_max_) obtained by the optical profilometer are presented in Table 2. Surface roughness decreased with time across IPR system, thickness, and grain-size groups. No overall significance of system was found using likelihood ratio tests (*P* = 0.07 for R_a_, *P* = 0.33 for R_z_, and *P* = 0.48 for R_max_).

**Table 2:** Surface roughness measurements (R_a_, R_z_, R_max_) at T0–T5 provided by the optical profilometer

	Surface roughness																	
	T0			T1			T2			T3			T4			T5		
IPR instrument	R_a_	R_z_	R_max_	R_a_	Rz	R_max_	R_a_	R_z_	R_max_	R_a_	R_z_	R_max_	R_a_	R_z_	R_max_	R_a_	R_z_	R_max_
DS-25	1.996	50.811	63.073	1.761	46.8	51.61	1.627	43.895	49.101	1.523	39.546	40.175	1.433	42.672	48.643	1.468	40.757	41.319
DS-40	2.706	77.553	80.898	2.272	60.146	71.697	2.318	53.886	55.318	2.069	54.128	64.354	1.868	49.4	55.217	1.841	52.39	60.024
DS-60	3.579	92.398	96.5	2.256	52.47	55.611	2.265	55.871	63.219	2.275	67.198	76.742	2.001	47.094	49.367	1.815	51.222	63.311
IS-25	2.123	46.556	49.907	1.265	36.031	42.811	1.094	24.967	35.24	1.002	21.14	27.393	0.958	21.296	28.162	0.886	18.024	31.953
IS-40	2.423	52.262	58.385	1.797	44.448	49.165	1.534	36.608	41.795	1.443	32.385	37.767	1.474	35.042	36.412	1.449	36.393	39.497
IS-60	3.639	77.174	77.914	2.82	70.49	75.396	3.115	64.874	72.997	2.866	60.143	57.158	2.821	70.393	84.167	2.638	57.729	64.94
SDC-15	1.469	37.944	41.337	1.179	28.534	37.355	0.939	20.882	28.813	0.866	24.548	37.364	0.798	18.176	35.35	0.964	29.739	39.506
SDC-20	2.046	49.054	46.547	1.351	32.965	41.713	1.26	31.506	36.009	1.14	39.034	47.517	1.089	27.566	30.763	1.083	32.601	43.837
SDC-30	2.962	88.329	102.899	2.394	60.385	60.518	2.106	59.55	60.958	1.859	63.778	68.31	1.774	57.301	67.678	1.74	57.731	74.252
OT-11	3.194	56.316	66.149	2.922	78.925	92.196	1.912	44.553	57.881	1.741	30.466	33.528	1.411	22.911	27.009	1.459	30.462	35.047
OT-13	2.609	37.305	39.579	2.551	40.367	43.287	2.064	36.415	40.861	2.087	42.491	55.831	2.517	88.871	126.988	2.205	36.562	39.991
OT-55	2.579	57.198	61.315	2.45	59.692	65.81	2.693	48.448	57.414	2.295	52.165	55.602	2.304	45.331	49.129	2.295	54.133	55.107
OT-56	3.108	53.304	57.259	3.045	57.001	59.035	2.669	48.473	57.826	2.65	56.407	62.212	2.787	61.525	68.337	2.75	47.704	50.539
OS-10	1.815	28.514	33.391	1.327	16.332	18.311	1.205	16.762	18.659	1.093	14.958	15.885	1.102	20.974	24.574	1.085	16.099	19.309
OS-20	2.994	61.357	64.217	2.548	59.923	55.016	2.538	61.091	69.381	2.284	55.479	60.335	2.162	54.391	72.695	2.105	53.419	61.681
OS-18	2.817	77.06	92.416	1.637	49.912	58.769	1.523	37.215	46.107	1.237	29.93	38.545	1.509	38.25	46.107	1.37	29.133	31.678

*DS* DiaStrip, *IS* Intensiv Ortho-Strips System, *SDC* G5-ProLign, *OT* Galaxy IPR Diamond Discs, *OS* OS Discs

There was a significant average decrease of R_a_, R_z_, and R_max_ for all systems for every unit increase in time by −0.171 μm (95% confidence interval [CI]: −0.203, −0.139; *P* < 0.001), −3.297 (95% CI: −4.493, −2.100; *P* ≤ 0.001), and −2.788 μm (95% CI: −4.422, −1.154; *P* = 0.001), respectively (Table 3). R_a_, R_z_, and R_max_ values increased significantly, i.e., by 0.194 μm (95% CI: 0.068, 0.321; *P* = 0.003), 5.890 μm (95% CI: 2.282, 9.497, *P* = 0.001), and 5.319 μm (95% CI: 1.258, 9.379; *P* = 0.010) as instrument thickness increased by one unit (Table 3). There was no significant average reduction of roughness values across grain sizes, viz. −0.008 μm (95% CI: −0.025, 0.008; *P* > 0.05), −0.244 μm (95% CI: −0.709, 0.221; *P* > 0.05), −0.179 μm (95% CI: −0.695, 0.337; *P* > 0.05) (Table 3).

**Table 3:** Coefficients, associated confidence intervals (95% CIs), and *P*-values from the random effects linear models for R_a_, R_z_, R_max_ by system, thickness, grain size group, and time

	R_a_				R_z_				R_max_		
System^a^	Coefficient	*P*-Value	95% CI	System^b^	Coefficient	*P*-Value	95% CI	System^c^	Coefficient	*P*-Value	95% CI
DS	0.181	0.505	−0.350, 0.712	DS	11.852	0.144	−2.851, 26.554	DS	12.263	0.141	−4.060, 28.587
IS	0.175	0.516	−0.353, 0.703	IS	5.022	0.500	−9.587, 19.632	IS	5.045	0.542	−11.175, 21.264
SDC	−0.491	0.109	−1.091, 0.109	SDC	−3.543	0.676	−20.153, 13.608	SDC	−0.428	0.964	−18.871, 18.014
OT	0.695	0.009*	0.172, 1.219	OT	11.195	0.130	−3.290, 25.680	OT	11.776	0.151	−4.305, 27.857
OS	Reference			OS	Reference			OS	Reference		
*Grain size*	−0.008	0.323	−0.025, 0.008	*Grain size*	−0.244	0.304	−0.709, 0.221	*Grain size*	−0.179	0.496	−0.695, 0.337
*Thickness*	0.194	0.003*	0.068, 0.321	*Thickness*	5.890	0.001*	2.282, 9.497	*Thickness*	5.319	0.010*	1.258, 9.379
*Time*	−0.171	0.000*	−0.203, −0.139	*Time*	−3.297	0.000*	−4.493, −2.100	*Time*	−2.788	0.001*	−4.422, −1.154

*DS* DiaStrip, *IS* Intensiv Ortho-Strips System, *SDC* G5-ProLign, *OT* Galaxy IPR Diamond Discs, *OS* OS Discs

^a^Overall significance of system for R_a_, *P* = 0.07

^b^Overall significance of system for R_z_, *P* = 0.33

^c^Overall significance of system for R_max_, *P* = 0.48

*Value statistically significant

**Fig. 1:**
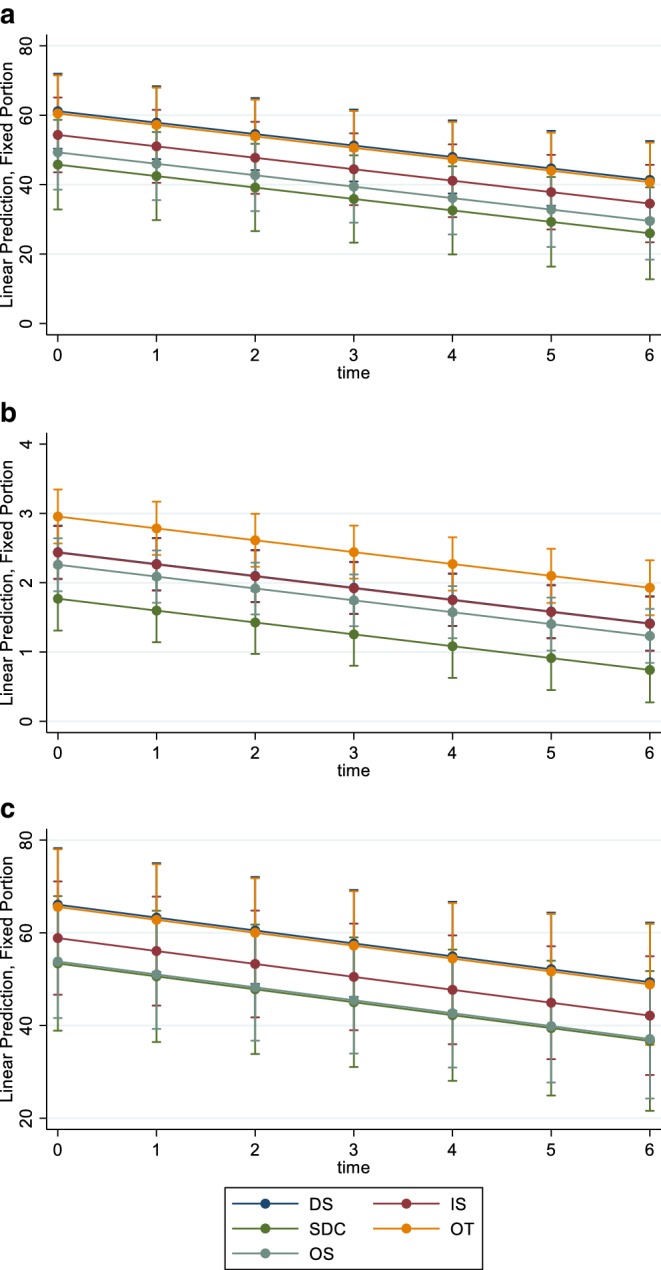
Roughness changes of interproximal reduction (IPR) instruments in relation to time (**a** R_a_; **b** R_z_; **c** R_max_). *DS* DiaStrip, *IS* Intensiv Ortho-Strips System, *SDC* G5-ProLign, *OT* Galaxy IPR Diamond Discs, *OS* OS Discs

There was no evidence of interaction between system and time as the likelihood ratio tests *P* values for R_a_, R_z_, and R_max_ were 0.88, 0.51, and 0.70, respectively, and thus the interactions terms were dropped from the model. Roughness reduction by time, was comparable among systems (Fig. 1).

## Discussion

As the popularity of IPR is increasing in nonextraction orthodontic treatment with fixed appliances and clear thermoplastic aligners, it is worthwhile to thoroughly explore the mechanical behavior of IPR systems. To the best of our knowledge, this is the first study designed to investigate the surface roughness changes in an extended list of commonly used handpiece-driven IPR instruments.

The lack of overall significance in roughness changes between systems indicates that no system was found superior to others in withstanding abrasive loss. All tested materials exhibited a significant reduction in surface roughness with time, which was comparable for all IPR systems. Given instrument surfaces were cleaned before each profilometric evaluation, it may be expected that in clinical conditions the decrease in roughness might be more rapid since besides detachment of diamond granules, increasing accumulation of tooth material on the instrument surface during the repeated applications might take place [16]. In addition, in daily practice, IPR is performed between adjacent teeth. In case proper contacts and mechanical access are not provided, forcing the stripping auxiliary into tight contact points and application of a heavy load by the clinician, will result in instrument deformation and a more rapid loss of abrasive power [16].

Thicker IPR auxiliaries showed significantly less abrasive wear compared to auxiliaries with thinner stripping segments. This finding implies that regardless of IPR system, thinner stripping instruments may require more frequent replacement when used in vivo. As other investigators stated, instrument thickness may influence the instrument deflection and achieved enamel reduction. The thicker or the more solid the IPR instrument, the more efficient the distribution of the applied force to the enamel surface [22].

Surface roughness of IPR systems was quantified in the present study by profilometry, a broadly used method for measuring the surface profile of dental materials [23–25]. Nevertheless, profilometry has been criticized for inducing sample damage and its inability to measure overall surface roughness due to scanning a single line in a preselected area [26, 27]. By using a noncontact optical profilometer, we avoided any potential sample damage. Although the profilometer used would allow measurement of the roughness parameters for whole surfaces, the different kinds of perforations of the auxiliaries made it impossible to measure surfaces in a standardized way for all the auxiliaries. Therefore, we decided to rather measure traces of maximal lengths across the cross-sections of the abrasive parts of the auxiliaries. Furthermore, the optical profilometer provides an extremely high vertical resolution (<10 nm) and a set of roughness values that permits statistical analysis [28].

It is well-accepted that the amount of enamel reduction is influenced by operator- or technique-related aspects such as exerted pressure, hardness, and particle size of the abrasive, IPR duration, and tooth-related aspects such as enamel hardness [17]. As there is no data in the literature about the optimal applied force [22], to ensure standardization of the experimental IPR technique, enamel preparation was carried out by a single clinician within a predefined period, strictly following manufacturers’ instructions for use.

Certain caveats need to be acknowledged when translating our study findings into clinical practice. The sample teeth were mounted in acrylic resin, and therefore, it may be presumed that no physiologic tooth movement during IPR was simulated. Alternative embedment in silicone, like in past studies [22, 29], has been criticized since silicone may fatigue faster that biological tissues. Possible loosening of the teeth in the silicone base could lead to insufficient resistance to the mechanical movement of the auxiliary, and eventually insufficient loading by the clinician during IPR [29]. Furthermore, during or after IPR in vivo, stretching of periodontal fibers might occur consequent to the initial aligning, causing tooth movement and underestimation of the stripping outcome [29]. Unlike clinical conditions, IPR in this in vitro investigation was carried out on individual teeth without the need for opening up the interproximal space. This was chosen deliberately to facilitate access to interproximal areas and direct study of surface roughness changes of IPR instruments after multiple applications.

Future studies should aim to evaluate the efficiency of powered IPR systems in vivo as well as user friendliness and patient comfort [22]. It would be useful to couple the abrasive wear of IPR auxiliaries with the actual amount of the stripped enamel, and to assess patient perception during IPR procedures with different systems. In this way, valuable recommendations can be made to clinicians about the lifecycle and frequency of replacement of IPR instruments to maximize treatment efficiency and patient comfort.

## Conclusions

No system was found superior to others in withstanding abrasive wear. All tested powered stripping materials presented a significant decrease of surface roughness after repeated in vitro use. The grain size of the stripping segment did not have a significant effect on the observed roughness changes. Significantly less abrasive wear was observed in thicker auxiliaries, implying longer potential clinical use compared to thin IPR auxiliaries.
